# Ms. Noemi Monika Szeifert

**DOI:** 10.1192/j.eurpsy.2022.1238

**Published:** 2022-09-01

**Authors:** N. Szeifert, S. Szilágyi, Á. Schmelowszky

**Affiliations:** 1 Peterfy Alexander Hospital, Crisis Intervention And Psychiatry, Budapest, Hungary; 2 University of Eötvös Lóránd, Clinical Psychology And Addictology, Budapest, Hungary

**Keywords:** COVID-19 Health Care Workers, Psychological distress, vicarious traumatization, PTSD

## Abstract

**Introduction:**

**Working in COVID – 19 Health Care Units – Psychological impacts (PTSD, Depression, Anxiety Disorders)** Working in the frontline during COVID-19 has put under extreme psychological and physical pressure the health care workers. The severe psychological symptomps can emerge on short, mid and long term as well. Our research is focusing on the psychological impacts of front line health care workers.

**Objectives:**

In our lecture, we summarize our reserach made in the National Traumatology Center, Budapest, Hungary among health care workers who worked in the front and in the second line during the most severe period of the pandemic in Hungary, and discuss probable risk factors for PTSD and chronic psychological distress related to COVID-19. Furthermore we make an overview on the most frequently used coping skills for dealing with the psychological stress caused by the pandemic among the health care workers. The sample taking was processed in July 2021, after 3 month the 3rd COVID – 19 wave officially ended in Hungary.

**Methods:**

123 health care workers completed the online survey anonimously, included Beck Depression Inventory, Spielberger Anxiety State Inventory, PTSD Checklist, Lazarus Coping Scale.

**Results:**

23% of HCW reported symptomps of depression, nearly 25% dealing with sleeping disorders and 38% with chronic fatigue, 42,50 % HCW suffering from anxiety disorders, 22% HCW experiencing symptomps of PTSD.

**Conclusions:**

With our results we would like to raise awareness of the challenges and severe psychological consequences that these colleagues, our national heroes we can say, are facing after working in COVID-19 Health Care Units.

**Disclosure:**

No significant relationships.

